# A novel fungal beta-propeller phytase from nematophagous *Arthrobotrys oligospora*: characterization and potential application in phosphorus and mineral release for feed processing

**DOI:** 10.1186/s12934-020-01346-9

**Published:** 2020-04-06

**Authors:** Xianjuan Hou, Zhen Shen, Na Li, Xiaowei Kong, Kangliang Sheng, Jingmin Wang, Yongzhong Wang

**Affiliations:** 1 School of Life Sciences, Anhui University, Hefei, 230601 Anhui China; 2 Key Laboratory of Human Microenvironment and Precision Medicine of Anhui Higher Education Institutes, Anhui University, Hefei, 230601 Anhui China; 3 Anhui Key Laboratory of Modern Biomanufacturing, Hefei, 230601 Anhui China; 4 Institute of Physical Science and Information Technology, Anhui University, Hefei, 230601 Anhui China

**Keywords:** Nematophagous fungi, *Arthrobotrys oligospora*, Phytase, Phosphorus and mineral release, Feed processing

## Abstract

Phytases are widely utilized in feed industry to increase the utilization of phosphorus, minerals, and amino acids for improvement of animal and human nutrition. At present, all known β-propeller phytases (BPP) have been generated from bacteria, particularly *Bacillus*. In this work we report for the first time a new fungal-derived BPP phytase. We identified a phytase highly differentially expressed only in the parasitic stage of a nematophagous fungus, *Arhtrobotrys oliogospora*, during the development of the 3D traps. We found that this phytase was homologous to the known bacterial BPP phytase, thus we referred the new phytase to Aophytase. The heterologous expression of codon-optimized Aophytase gene in *Pichia pastoris* was successfully investigated to yield recombinant Aophytase (r-Aophytase) with high specific enzyme activity of 74.71 U/mg, much higher than those of recombinant BPP phytases derived bacteria. The kinetic parameters of the r-Aophytase, the optimum pH and temperature, as well as the effects of surfactant, EDTA and different ions on its enzyme activity were further investigated. The potential utilization of r-Aophytase in feed processing was finally explored. We found that the optimal pH value was about 7.5, and the optimal temperature was 50 °C.; r-Aophytase significantly increased the release of inorganic phosphorus from soybean meal, and improved the release of soluble minerals from the durum wheat flour and finger millet flour. The findings indicate its potential utilization in the feed processing to ameliorate nutritional value of cereals and animal feed in the future.

## Introduction

Phytases are widely used in feed industry to increase the utilization of phosphorus, minerals, and other nutrients such as protein, starch and fat [1–3]. The diversity and abundance of phytases in nature were widely reported [4]. Plants, including rice, wheat, corn, soybeans and other plant seeds, are traditionally important sources for phytase production [5]. Presently, microbes, such as bacteria, yeast, and filamentous fungi are major sources for the discovery and production of novel phytases. Owing to the excellent physical and chemical properties of known fungal phytases, filamentous fungi, especially *Aspergillus spp*., are promising source of the development of novel commercial phytases [6].

According to their protein structures and catalytic mechanisms, phytases include the following categories: histidine acid phosphatase phytase (HAP) [7], β-propeller phytase (BPP) [8], purple acid phosphatase phytase (PAP) [9] and cysteine phosphatases (CP) [10]. Of these categories, BPP phytases is a new type of alkaline phosphatase enzymes recently reported. It is the most abundant and structurally diverse type of phytases in nature. To date, all known BPP phytases have been generated from bacteria, particularly Bacillus [10]. For example, the phytase phyC isolated from *Bacillus subtilis* and TS-Phy from *B. amyloliquefaciens* belong to BPPs. Both showed a similar structure of six-bladed propeller [8], and Ca^2+^ dramatically affected their catalytic activities and thermal stabilities [11]. In general, BPPs have two phosphate binding sites [12] that are capable of hydrolyzing phytic acid to inositol triphosphate (IP3). To the best of our knowledge, the currently known fungal-derived phytases were characterized as HAP phytases [10], and few fungal-derived BPP phytases were reported.

*Arthrobotrys oligospora* is an important nematophagous fungus, widely distributed in various soil environments [13]. In its asexual stage, *A. oligospora* has two typical phases, i.e., saprophytic phase and parasitic phase. Upon nematodes or proteinous substances are present in their growth environment, *A. oligospora* swifts from saprophytic to parasitic phase, in which heavy 3D traps are developed and matured. These 3D traps adhere to, penetrate, fix, and digest the nematodes to provide nitrogen nutrition for their growth in a low nutrient environment [14]. Due to its unique ability to trap nematodes, the fungus has been widely used for biological control of parasitic nematodes in plant and animal [13]. At present, the key molecular events and the specific components of surface polymers of the 3D traps still remain elusive. Tunlid et al. and Wang et al. proposed that proteinous and carbohydrate polymers on these 3D surface play the key role in the process of capturing nematodes [15, 16], by which *A. oligospora* is considered a valuable resource for exploration of enzymes with novel properties.

This work here reports for the first time a new fungal-derived BPP phytase. We identified a phytase highly differentially expressed only in the parasitic stage of *A. oliogospora* during the development of the 3D traps. We found that this phytase was found to be homologous to the known bacterial BPP phytase, thus we referred the new phytase to Aophytase. In this paper, the recombinant expression of Aophytase, the enzymatic activity and kinetic parameters of the recombinant Aophytase (r-Aophytase), the optimum pH and temperature, as well as the effects of surfactant, EDTA and different ions on its enzyme activity were investigated. We found that the fungal-derived Aophytase had a high specific enzyme activity and effectively improved the release of inorganic phosphorus and soluble minerals from different feedstuffs, suggesting its potential uses in feed processing in the future.

## Materials and methods

### Strain, media and chemicals

*Arthrobotrys oligospora* (ATCC 24927) was purchased from the American Type Culture Collection (Manassas, VA, USA). Soya peptone broth (0.01%) was prepared by dissolving 0.2 g soya peptone in 2000 mL ddH_2_O, supplemented with 0.005% Val and 0.005% Phe, or supplemented with 3 mM (NH_4_)_2_SO_4_. Chaotropic LiCl solution consists of 5 M LiCl and 10 mM Tris–HCl (pH 7.4). Five mmol/L of standard phosphorus stock solution was prepared by dissolving 0.68 g KH_2_PO_4_ in 1000 mL ddH_2_O. The AMES solution was prepared by mixing 10% ascorbic acid with 0.42% ammonium molybdate-1 mol/L sulfuric acid at ratio of 1:6 (v/v).

### Collection and MS analysis of surface proteinaceous biopolymer from *A. oligospora* with parasitic 3D traps

Fifteen mL of *A. oligospora* conidia at the density of 1.0 × 10^6^/mL was inoculated to 2 L of 0.01% soya peptone broth in 5-L-fermenter (Bxbio, Shanghai). Under 4 L/min of oxygen flow, the inoculated conidia were cultured at 25 °C for 5 days [17]. To induce the development 3D traps, the soya peptone broth supplemented with 0.005% Val/Phe was used [18]; to inhibit the formation of 3D traps, the soya peptone broth supplemented with 3 mM (NH_4_)_2_SO_4_ was accordingly used [19].

After 5 days of fermentation, the mycelia were collected by filtration. The collected mycelia were washed 5 times with 10 mL of pre-cooled ddH_2_O, the excess water of the mycelia was absorbed, and 10 mL of chaotropic LiCl solution was added and incubated at 4 °C for 10 min in a shaker at about 40 rpm [15]. The extract was concentrated by ultrafiltration and was conducted for SDS-PAGE analysis. Five differentially expressed protein bands excised from the gel were hydrolyzed by trypsin and separated by C18 reversed phase capillary column of LC with mobile phase comprise of 0.1% formic acid and 100% acetonitrile, followed by mass spectrometry analysis in a positive ion mode.

### Sequence analysis and homologous modeling

With human Wnt inhibitor (Wif, c2ygqA), Bacillus-derived 3-phytase (d1h6la), human sputum factor interacting protein (Hhip, c3ho5B), and human Wnt signaling co-receptor LRP6 (c3soqA) as templates, homologous modeling of Aophytase was conducted by using the Phyre2 online tool (http://www.sbg.bio.ic.ac.uk/phyre2/html/page.cgi?id=index) in the intensive modeling mode. Meanwhile, Clustal X2 and Bioedit analysis software were used to align the amino acid sequence of Aophytase with those of the above templates, and to analyze the conserved amino acids in Aophytase.

### Codon optimization and recombinant expression of Aophytase in *Pichia pastoris*

According to the amino acid sequence of Aophytase (Genbank accession NO. 22896793), the codon was optimized for *P. pastoris* expression system. The optimized gene fragment (Genbank accession NO. MN688990) was then synthesized, digested with *EcoR*I and *Sal*I, and ligated to vector pPICZαA at 16 °C overnight. The ligation mixture was transformed into competent DH5α cells in LB/Zeocin agar plate. Positive transformants were then identified to prepare the expression vector pPICZαA-phytase. Tweenty μg of pPICZαA-phytase was then linearized by *Sac*I, and 10 μL of the linearized plasmid was added into 100 μL of the competent cells of yeast GS115 in a 0.2 cm ice-cold cuvette. After 5 min incubation in ice bath, the yeast was transformed by ligation solution via electroporation (200 Ω, 1.5 kV, 25 μF). 500 μL of 1 M sorbitol solution was immediately added to the cuvette, and incubated for 20 min at room temperature. The mixture was finally added to 500 μL YPD medium, and incubated at 30 °C for 1 h in a shaker at 220 rpm. 100 μL of the above mixture was spread in the YPDSZ plate, and cultured at 30 °C for about 2–3 days. The positive transformants were identified by colony PCR using primers 5′AOX (5′-GACTGGTTCCAATTGACAAGC-3′) and 3′AOX (5′-GCAAATGGCATTCTGACATCC-3′). Twenty clones were selected from YPDSZ plates for PCR verification. The PCR reaction conditions were as follows: pre-denaturation at 95 °C for 3 min; denaturation at 95 °C for 20 s, annealing at 55 °C for 20 s, extension at 72 °C for 1 min for a total of 30 cycles; and final extension at 72 °C for 10 min.

The recombinant Aophytase (r-Aophytase) was finally inducible expressed in the genetically engineered *Pichia pastoris*. The engineered *Pichia* strain was inoculated into 50 mL of BMGY medium and cultured at 30 °C in a shaker at 220 rpm for about 48 h. Once the OD_600_ reached about 2–6 (~ 4.6), the culture was centrifuged at 1500 rpm for 10 min. The resultant cell pellet was resuspended in 200 mL of BMMY medium, and was cultured at 30 °C in a shaker of 220 rpm for 72 h. The induced culture was then centrifuged at 8000 rpm for 10 min at 4 °C. The supernatant was collected as a crude enzyme solution and was finally analyzed by SDS-PAGE.

### Purification of r-Aophytase

Five mL of Ni–NTA beads 6FF was added to the collected crude enzyme solution and incubated at 4 °C for 2 h. The incubated beads were loaded to an empty column, and the flow-through was collected. The column was first eluted with 20 mM imidazole buffer (10 mM Na_2_HPO_4_, 2 mM KH_2_PO_4_, 0.8% NaCl, 0.02% KCl, 5% Glycerol, 20 mM imidazole, pH 6.0), followed by elution with 100 and 250 mM imidazole buffer. Finally, the collected eluates were all dialyzed in an imidazole-free buffer (10 mM Na_2_HPO_4_, 2 mM KH_2_PO_4_, 0.8% NaCl, 0.02% KCl, 5% Glycerol, pH 6.0), and analyzed by SDS-PAGE and Western blotting.

### r-Aophytase activity assay

One mL of the phosphorus standard assay solution at the final concentration ranging from 0.1 to 0.5 mmol/L was transferred to 15 mL centrifuge tubes, followed by the addition of 1 mL AMES solution. After incubation at 50 °C in water bath for 20 min, the OD values of the samples were measured at λ_700_nm. The OD values are then plotted versus phosphorus concentration to yield a standard curve for phosphorus determination.

Based on the standard curve, the r-Aophytase activity assay was determined and calculated. Briefly, 100 μL of the diluted enzyme solution at 8.8 µg/mL and 900 μL of substrate solution (2 mM sodium phytate in 100 mM Tris–HCl buffer, pH 7.0) were mixed, and then incubated at 37 °C for 30 min. The reaction was immediately stopped by adding 1 mL of 10% trichloroacetic acid, followed by the addition of 2 mL AMES solution, and measurement of the absorbance at λ_700_ nm [20]. The experiment was performed in triplicate.

The enzyme activity unit (U) of r-Aophytase is defined as the amount of enzyme required to release 1 μmol of inorganic phosphorus per minute from sodium phytate at 37 °C [21]. The enzyme activity (μmol min^−1^) and specific enzyme activity (μmol min^−1^mg^−1^) are calculated following the Eqs. (1) and (2), respectively.

1 $$ {\text{Enzyme activity }} = \, \left[ {\left( {{\text{OD}} - {\text{OD}}_{0} } \right) \times {\text{N}}} \right] /\left( {{\text{K}} \times {\text{T}}} \right) $$

2 $$ {\text{Specific enzyme activity }} = {\text{ enzyme activity }}\left( {\text{U}} \right)/{\text{ amount of enzyme }}\left( {\text{mg}} \right) $$

where OD is the absorbance of the sample measured at λ_700_ nm; OD_0_ is the absorbance of the control sample measured at λ_700_ nm; N is the dilution factor of the sample; K is the slope of the standard curve; T is enzymatic reaction time.

### Effect of pH, temperature, metal ions, surfactant and chelating agent on r-Aophytase activity

Effects of pH [22]. Two mM sodium phytate solution was prepared in different pH buffers (100 mM), including glycine–HCl (pH 2.0–3.5), sodium acetate-acetic acid (pH 3.5–6.5), Tris–HCl (pH 6.5–9.0) and glycine–NaOH (pH 9.0–10). Nine hundred μL of sodium phytate solution and 100 μL of the diluted r-Aophytase solution were mixed, and then incubated at 37 °C for 30 min. The OD_700_ values of the above reaction mixtures were measured. The experiment was performed in triplicate. The relative enzymatic activities are plotted versus pHs to yield a response curve for optimal pH of the enzyme.

Effects of temperature [23]. Two mM sodium phytate was prepared in 100 mM Tris–HCl buffer (pH 7.0). Nine hundred μL of sodium phytate solution and 100 μL of the diluted r-Aophytase solution were mixed, and then incubated at various temperatures ranging from 20 to 70 °C at intervals of 10 °C. The OD_700_ values of the above reaction mixtures were measured. The experiment was performed in triplicate. The relative enzymatic activities are plotted versus temperatures to yield a response curve for optimum temperature of the enzyme.

Effects of metal ions, surfactants and complexing agents [24]. Two mM sodium phytate was dissolved in 100 mM Tris–HCl buffer (pH 7.0), followed by the addition of 1 mM Ca^2+^, Na^+^, K^+^, Li^+^, Mg^2+^, Fe^3+^, Ni^2+^, Cu^2+^, Zn^2+^, Mn^2+^, SDS and EDTA to prepare the reaction solution. Nine hundred μL of the reaction solution and 100 μL of the diluted r-Aophytase solution were mixed, respectively, and then incubated at 37 °C for 30 min. The OD values in the above reaction solutions were measured. The experiment was performed in triplicate.

### Determination of kinetic constants of r-Aophytase

Nine hundred of sodium phytate solutions at the concentration ranging from 0.125 to 4.0 mM were prepared in 100 mM Tris–HCl (pH 7.5), followed by the addition of 100 μL of r-Aophytase solution (0.5U). The reaction mixtures were fully mixed and incubated at 50º C for 10 min. The reactions were stopped by the addition of 1 mL of 10% trichloroacetic acid, followed by the addition of 1 mL AMES solution and incubation at 50 °C in water bath for 20 min. The OD_700_ values of the above reaction mixtures were measured. The experiment was performed in triplicate. The kinetic constants were calculated using the Lineweaver–Burk method [25].

### Hydrolysis of phytate phosphorus in soybean meal with r-Aophytase

The phosphorus release from soybean meal after digestion of different amounts of r-Aophytase (0.5 U–2.5 U) was measured [26]. One gram of soybean meal was dispersed in 9 mL of 100 mM Tris–HCl (pH 7.5) in 50 mL centrifuge tubes, followed by incubation at 37 °C for 60 min in a shaker of 150 rpm. Then, 1 mL of r-Aophytase was added to the suspension of soybean meal to give a final amount of 0.5–2.5 U/g of soybean meal. The reaction mixtures were incubated at 50 °C for 10 min, and stopped by the addition of 1 mL of 10% trichloroacetic acid. 1 mL AMES solution was finally added and incubated at 50 °C in water bath for 20 min. The OD_700_ values of the above reaction mixtures were measured. The experiment was performed in triplicate.

### Mineral release from durum wheat and finger millet flour

The total amount of minerals was first determined according to the procedure as previously described [26]. One gram of durum wheat and finger millet flour was dispersed in 5 mL of ddH_2_O, and was incubated at 50 °C for 2 h, respectively. Six mL of the mixed acids, i.e. nitrogen/sulfuric acid/perchloric acid at ratio of 10/1/4 (v/v/v), was added to the above suspension. The samples were dried at 200 °C in a fume hood, followed by dried in a muffle furnace at 450º C until the samples turned white powder. The powder was then dissolved in 1 mL of 35% HCl, and replenished to 10 mL with ddH_2_O. The samples were filtered, diluted 100-fold, and applied to ICP-MS analysis. The tests were done in triplicate for each sample.

The released minerals was then determined following the previous procedure [26]. One gram of durum wheat flour and finger millet flour was dispersed in 6 mL buffer (140 mM NaCl and 5 mM KCl), respectively. Twenty U of r-Aophytase was added to each suspension, and replenished to 10 mL with ddH_2_O. The reaction mixtures were incubated at 50 °C for 2 h, followed by centrifugation at 5000 rpm for 20 min. The supernatants were diluted 50 times for ICP-MS measurement. The tests were done in triplicate for each sample. The changes in release ratios of soluble minerals after r-Aophytase treatment were calculated according to the Eq. (3).

3 $$ {\text{Changes in release ratios }}\% \, = \, \left( {{\text{A }}{-}{\text{ C}}} \right) /{\text{ T}} \times 100\% $$

where A (μg) is the released amount of soluble minerals after the r-Aophytase treatment; C (μg) is the released amount of soluble minerals without the r-Aophytase treatment, i.e. control; T (μg) is the total amount of minerals in feedstuff.

## Results

### Main surface proteins from *A. oligospora* with parasitic 3D traps determined by MS sequencing

**Fig. 1:**
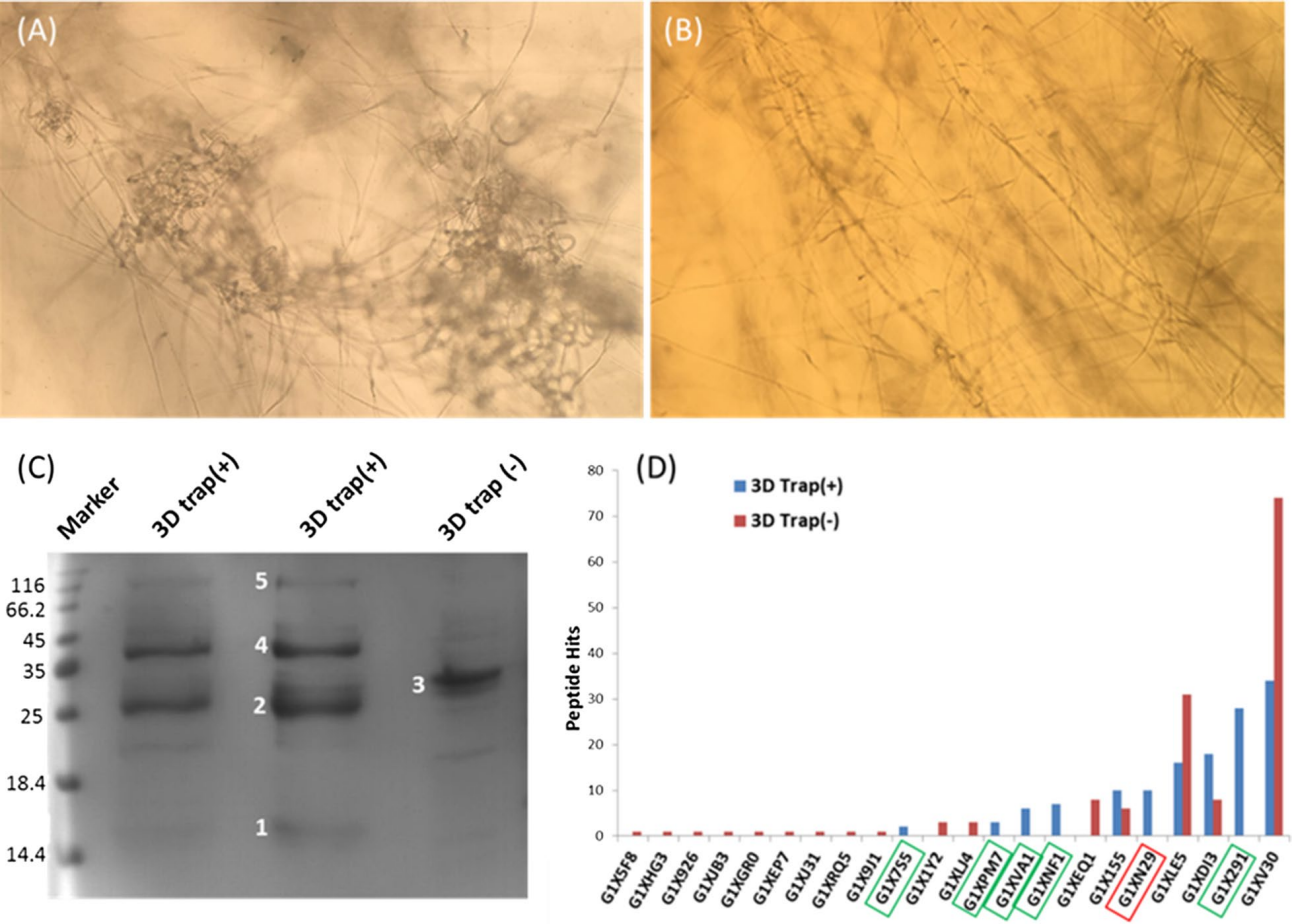
Main surface proteins from *A. oligospora* with parasitic 3D traps were determined by SDS-APGE and MS sequencing. **a** In the soya peptone-Val/Phe broth, heavy 3D traps were produced after cultured at 25 °C for 5 days with 4 L/min of oxygen flow; **b** in soya peptone-(NH_4_)_2_SO_4_ broth, no 3D traps were observed under the same culture conditions; **c** SDS-PAGE analysis for the surface proteins from both the mycelia with 3D traps, 3D Trap(+), and the mycelia without 3D traps, 3D Trap(−), extracted by using chaotropic LiCl solution; **d** mass spectrometry sequencing results for the major protein bands 1–5 from (**c**). Red histogram represents the 3D Trap(−) sample; Blue histogram represents the 3D Trap(+) sample. Rectangular boxes represent the unique proteins only expressed in the 3D Trap (+) sample. Red rectangular box: G1XN29 protein, i.e. Aophytase

In order to analyze the surface proteins of the 3D traps of *A. oligospora*, we successfully cultured the mycelia producing heavy 3D traps, i.e. 3D Trap(+), and the mycelia without 3D traps, i.e. 3D Trap(−), in a 5L small fermenter by adequate oxygenation. In soya peptone-Val/Phe broth, abundant 3D traps were produced after cultured at 25 °C for 5 days with 4 L/min of oxygen flow (Fig. 1a); while in soya peptone-(NH_4_)_2_SO_4_ broth, no 3D traps were observed under the same culture conditions (Fig. 1b).

The surface proteins of both the mycelia were successfully extracted by using chaotropic LiCl solution. SDS-PAGE analysis showed that the surface proteins were completely different between the 3D Trap (+) and 3D Trap (−). As shown in Fig. 1c, the 3D Trap(+) sample has 4 major protein bands, referred to bands 1, 2, 4, and 5, respectively; while the 3D Trap(−) has only one major protein band, referred to band 3. We further applied the 5 protein bands for mass spectrometry sequencing. In comparison with the peptide hits of different sequenced proteins, we found both the 3D Trap(+)and the 3D Trap(−) samples contained 22 different proteins, and that at least 6 completely different proteins were only expressed in the former (Fig. 1d). Among the six differently expressed proteins, G1XN29 protein is fungal-derived phytase, referred to Aophytase in this work.

### Multiple sequence alignment and homology modeling

**Fig. 2:**
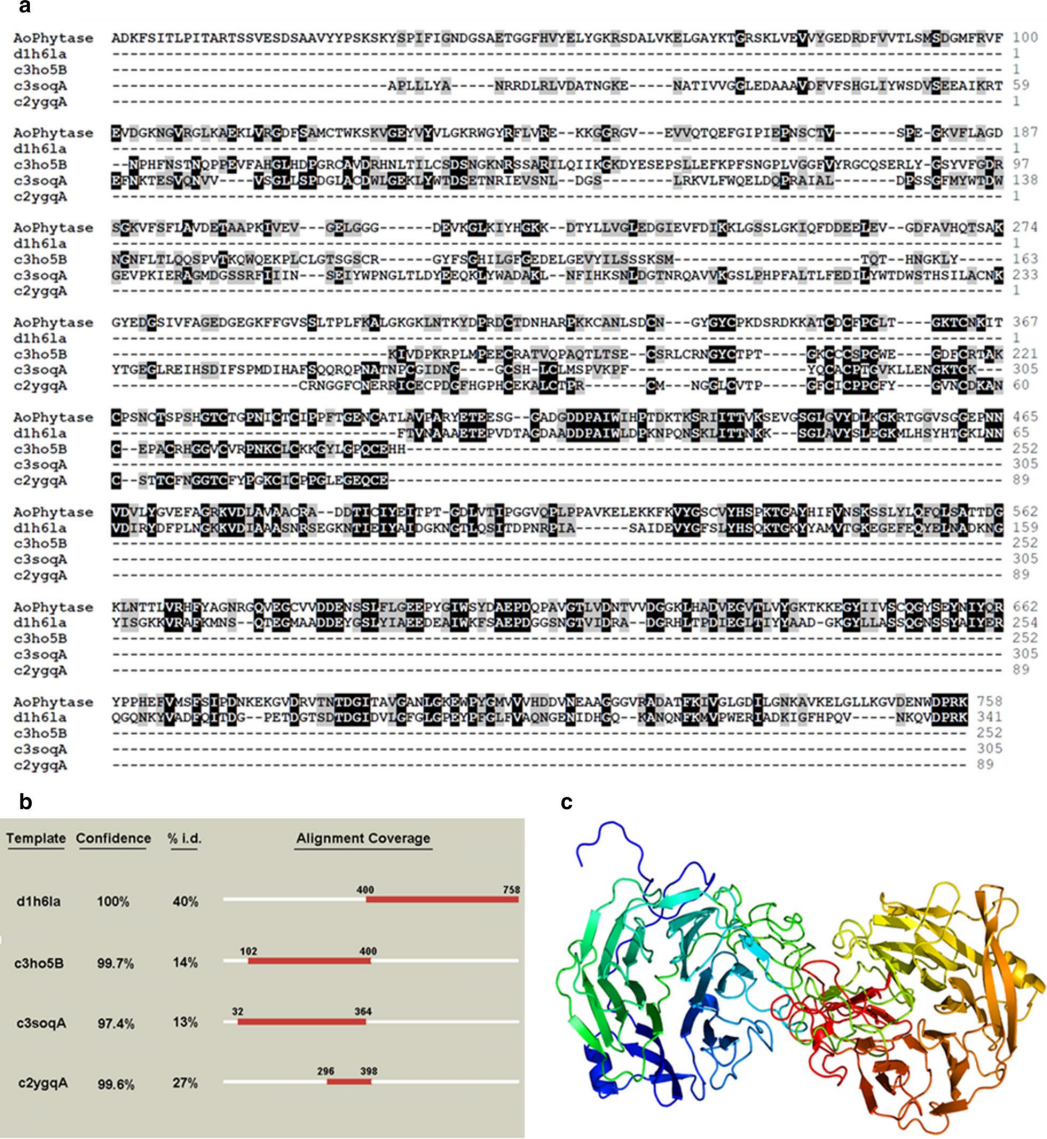
Multiple sequence alignment and homologous modeling of Aophytase. **a** Alignment of Aophytase with d1h6la, c3ho5B, c3soqA, and c2ygqA. **b** Using d1h6la, c3ho5B, c3soqA, and c2ygqA as templates, homolog confidence, sequence identity (% i.d.), and alignment coverage of each template versus Aophytase were compared. **c** Homologous modeling of Aophytase by using d1h6la, c3ho5B, c3soqA, and c2ygqA as templates

To explore the structure and function of Aophytase, we used the online homology modeling program, Phyre2, to select appropriate templates for multiple sequence alignment and homology modeling. We found that Aophytase was in a certain degree homologous with bacillus-derived 3-phytase (1h6l), human sonic hedgehog interacting protein Hhip (3ho5), human Wnt signaling co-receptor LRP6 (3soq), and human Wnt inhibitor Wif (2ygq) with the identity of 40%, 14%, 13%, and 27%, respectively (Fig. 2b). Aophytase was aligned with the amino acid sequence of d1h6la, c3ho5B, c3soqA, and c2ygqA (Fig. 2a), showing that they correspondingly covered Aophytase with different domains, with coverage rates of 47%, 39%, 43%, and 13%, respectively. Homologous modeling of Aophytase was then performed by using d1h6la, c3ho5B, c3soqA and c2ygqA as templates. We found that Aophytase contained two β-propeller domains and two EGF-like domains. The N-terminal β-propeller domain was homologous with Hhip (c3ho5B) and LRP6 (c3soqA), while the C-terminal β-propeller domain was homologous to the 3-phytase (d1h6la) derived from *Bacillus*. The middle EGF-like domain has higher homology with the Wif (c2ygqA).

### High expression of recombinant Aophytase in *Pichia pastoris*

**Fig. 3:**
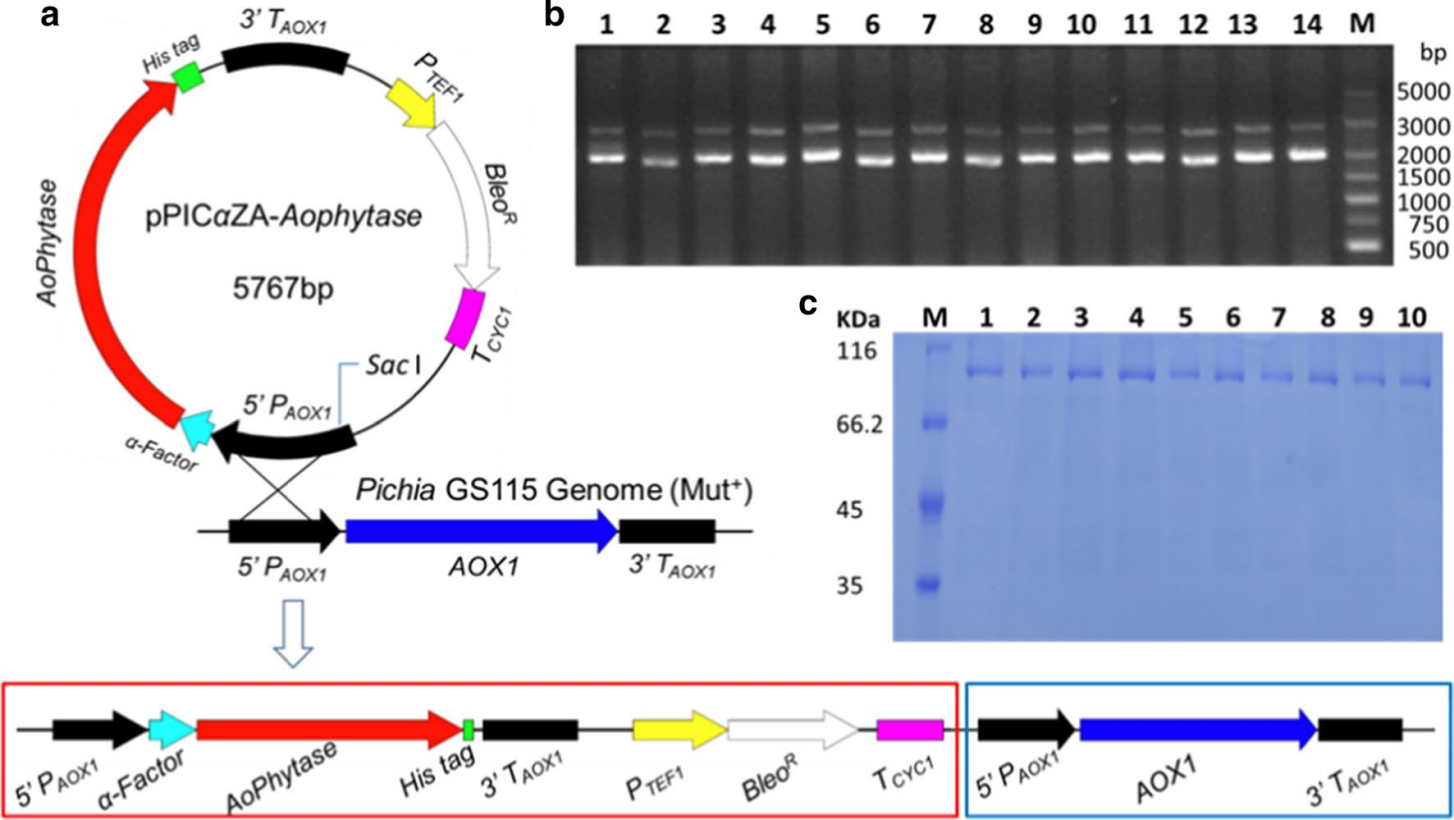
Integration of codon-optimized gene of Aophytase in the *Pichia* genome and its inducible expression. **a** Schematic diagram of integration of linearized pPICZαA-phytase in the yeast genome; **b** PCR identification of positive transformants picked from bleomycin selection plates; **c** SDS-PAGE analysis of recombinant proteins in fermentation broth of the positive transformants induced with methanol

The pPICZαA-phytase expression plasmid was successfully constructed by using codon-optimized Aophytase gene (Additional file 1: Fig. S1), followed by linearization of pPICZαA-phytase with SacI enzyme (Additional file 1: Fig. S2). *Pichia pastoris* GS115 was transformed by the linearized pPICZαA-phytase, resulting in that the Aophytase gene and corresponding screening gene expression cassette, *ble*, were integrated into 5′ promoter region (5′PAOX1) of the AOX 1 gene in *Pichia pastoris* GS115 genome by a single crossover homologous recombination mechanism (Fig. 3a). Twenty transformants were picked from the bleomycin selection plate for PCR verification, demonstrating that they all are positive transformants (Fig. 3b). Ten positive transformants were then randomly selected and induced for 72 h with methanol for recombinant expression of Aophytase. Figure 3c shows the result of SDS-PAGE analysis of the concentrated fermentation supernatant, and we found that there is protein band with MW of 66.2–116 KD, indicating that recombinant Aophytase could be effectively induced in the genetically engineered yeast by methanol and the r-Aophytase was secreted into the culture medium.

### Purification of r-Aophytase and western blotting

**Fig. 4:**
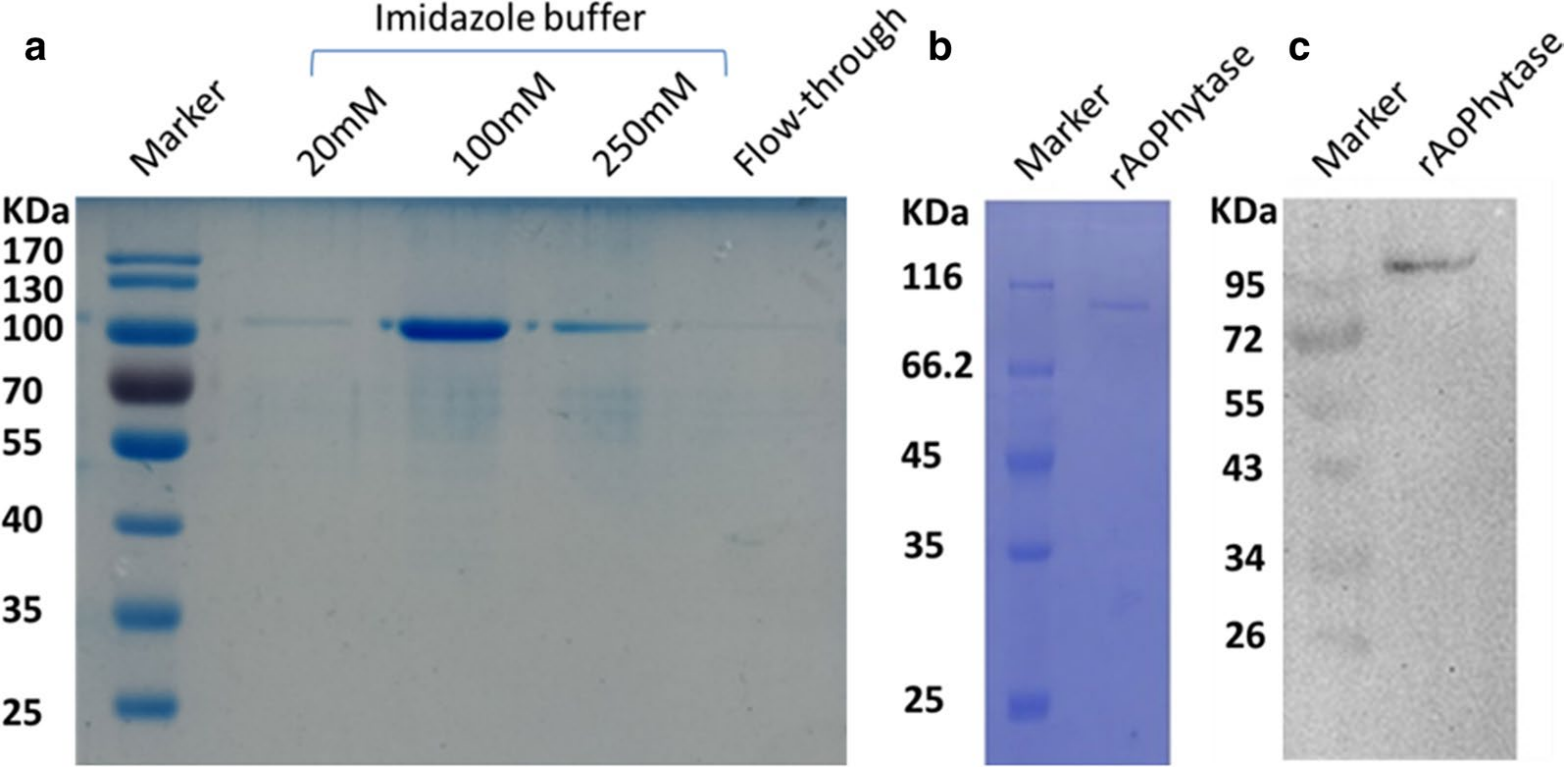
Purification of r-Aophytase by Ni NTA agarose affinity chromatography and its SDS-PAGE and western blotting analysis. **a** The r-Aophytase fraction was obtained by Ni NTA agrose affinity chromatography following elution with 100–250 mM imidazole buffer; **b** SDS-PAGE analysis of the purified r-Aophytase; **c** Western blotting of the purified r-Aophytase

Two hundred mL of the crude enzyme solution was further purified by Ni NTA agarose affinity chromatography. SDS-PAGE analysis showed that the purified r-Aophytase was obtained following elution with 100–250 mM imidazole buffer (Fig. 4a). After combining both the fractions eluted from 100 and 250 mM imidazole buffer, the purified r-Aophytase sample was dialyzed against an imidazole-free buffer. SDS-PAGE and western blotting analysis showed that it was electrophoretically pure and had a molecular weight between 95 and 116 kD (Fig. 4b, c). The molecular weight of r-Aophytase successfully expressed in *Pichia pastoris* is slightly larger than the theoretical molecular weight of Aophytase (84.2 KD), presumably resulting from the result of post-translational modification of glycosylation.

### Catalytic properties of recombinant Aophytase

**Fig. 5:**
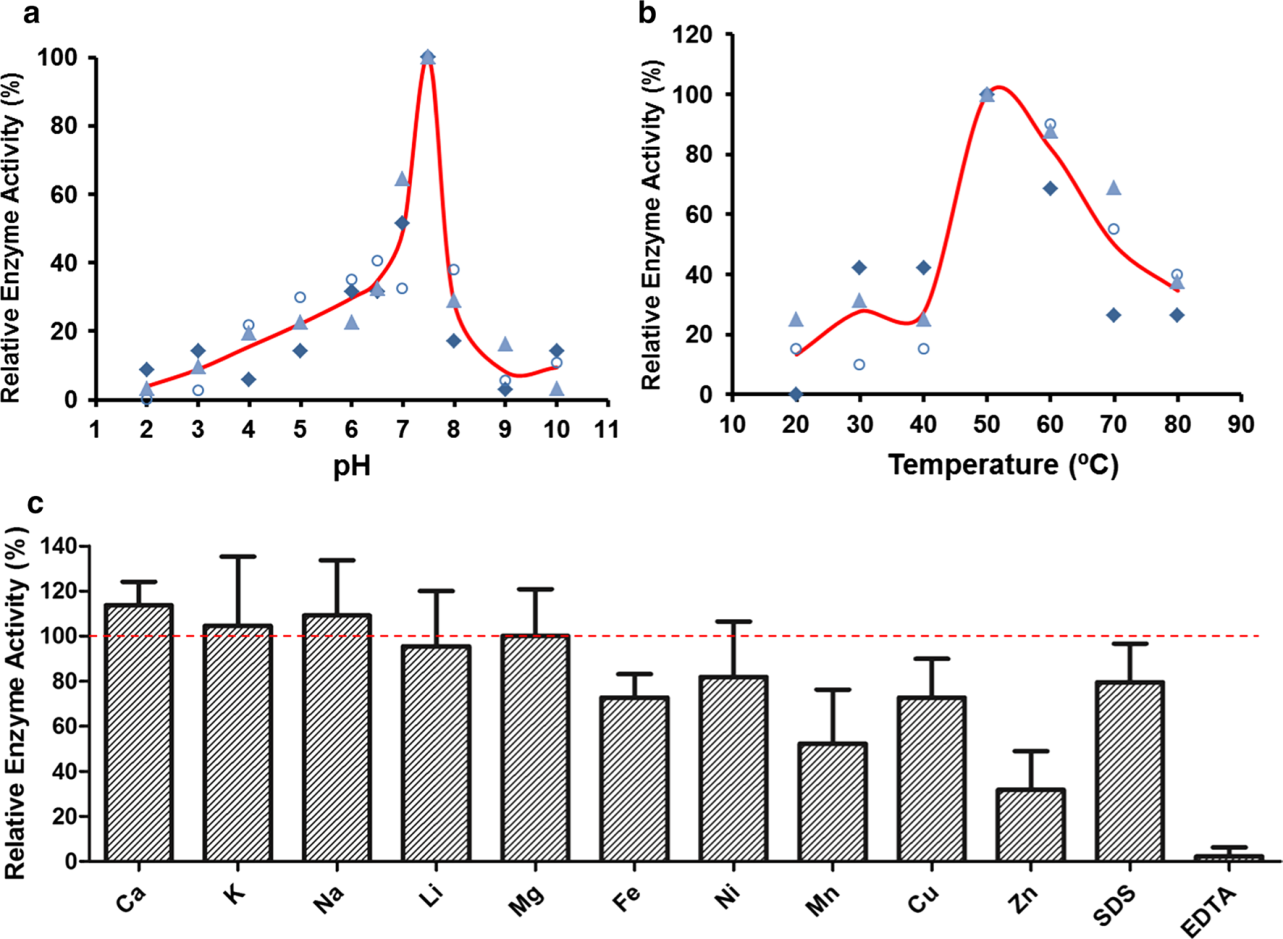
Effects of pH, temperature, surfactant, chelating agent and metal ions on the activities of r-Aophytase. **a** The activity of the r-Aophytase as a function of pH; **b** the activity of the r-Aophytase as a function of temperature; **c** the effects of surfactant, chelating agent and different metal ions on r-Aophytase activities

According to the standard curve of phosphorus (Additional file 1: Fig. S3), the specific enzyme activity of r-Aophytase was determined to be 74.71 U/mg (Table 1). The activity of the r-Aophytase as a function of pH is shown in Fig. 5a. It shows significantly high phytase activity between pH 5–8 with an optimal pH of 7.5. This property generally agrees with alkaline BPP phytase. The changes in the activity of the r-Aophytase in the temperature range of 20–80 °C were observed in Fig. 5b, showing that the optimal temperature is 50 °C. Furthermore, Fig. 5c shows the effect of surfactant SDS, chelating agent EDTA and different metal ions on r-Aophytase activity. We found that Ca^2+^, Na^+^, K^+^ slightly enhanced the enzyme activity, and Li^+^ and Mg^2+^ slightly inhibited the enzyme activity, while Fe^3+^, Ni^2+^, Cu^2+^, Zn^2+^, Mn^2+^, SDS and EDTA had obvious inhibition effect on the enzyme activity in certain degrees. Among them, EDTA, Zn^2+^ and Mn^2+^ had the most obvious inhibitory effects on their activities, and the inhibition rates reached 47.7%, 68.2% and 97.7%, respectively.

**Table 1:** Comparison of the specific activity of r-Aophytase with bacterial-derived recombinant BPP phytases

Phytase resources	Expression stains	Phytase activity	Refs.
*A. oligospora*	*P. pastoris*	71.74 U mg^−1^	This work
*Bacillus* sp. YCJS	*E. coli* BL21	14 U mg^−1^	[35]
*B. subtilis* ARRMK33	*Rosetta gami* B DE3	15.3 U mg^−1^	[36]
*Janthinobacterium* sp. TN115	*E. coli* BL21	21 U mg^−1^	[21]
*Pedobacter nyackensis* MJ11 CGMCC 2503	*E. coli* BL21	0.48 U mL^−1^	[22]
*B. licheniformis* ZJ-6	*P. pastoris*	0.23 U mL^−1^	[34]
*Serratia* sp. TN49	*E. coli* BL21	2.07 U mg^−1^	[24]

### Enzymatic kinetics of r-Aophytase

**Fig. 6:**
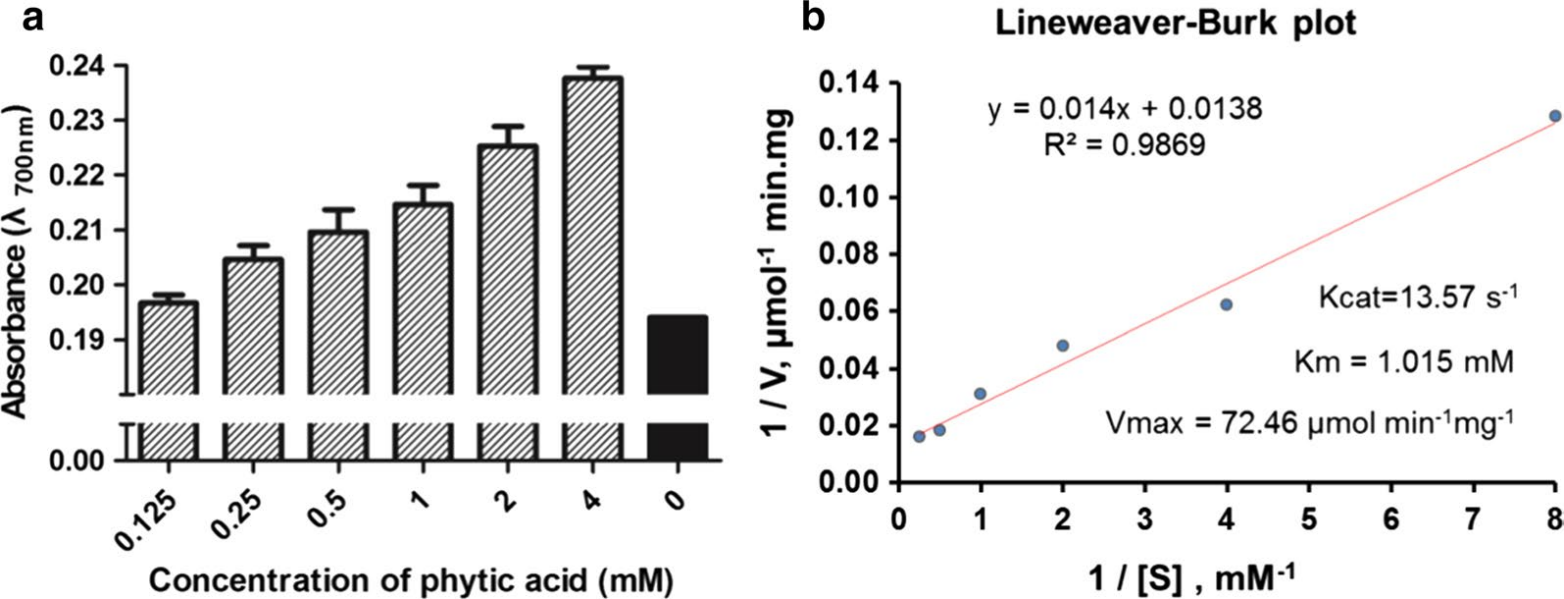
r-Aophytase enzyme kinetic parameters. **a** The released inorganic phosphorus gradually increased with an increase in the concentration of its substrate, phytic acid, when the enzyme amount kept constant. **b** Lineweaver–Burk plot and the corresponding fitting equation: y = 0.014 + 0.0138. Based on this equation, 72.46 μmol min^−1^ mg^−1^, K_m_ = 1.015 mM, and K_cat_ = 13.57 s^−1^ were obtained

To calculate its kinetic parameters, the specific enzyme activities of r-Aophytase were determined under optimal reaction conditions, i.e. pH 7.5 and 50 °C. We found that the released inorganic phosphorus gradually increased with an increase in the concentration of its substrate, phytic acid, when the enzyme amount kept constant (Fig. 6a). The reciprocal of the reaction rate V, 1/V, is then plotted on the ordinate as a function of the reciprocal of the substrate concentration 1/[S], plotted on the abscissa. As shown in Fig. 6b, Lineweaver–Burk plot and the corresponding fitting equation, y = 0.014 + 0.0138, are obtained. Based on the equation, V_max_ is calculated as 72.46 μmol min^−1^mg^−1^ and K_m_ as 1.015 mM; according to theoretical Mw of 84.195 kDa, K_cat_ of r-Aophytase is calculated as 13.57 s^−1^.

### r-Aophytase dramatically improve release of phosphorous and minerals

**Fig. 7:**
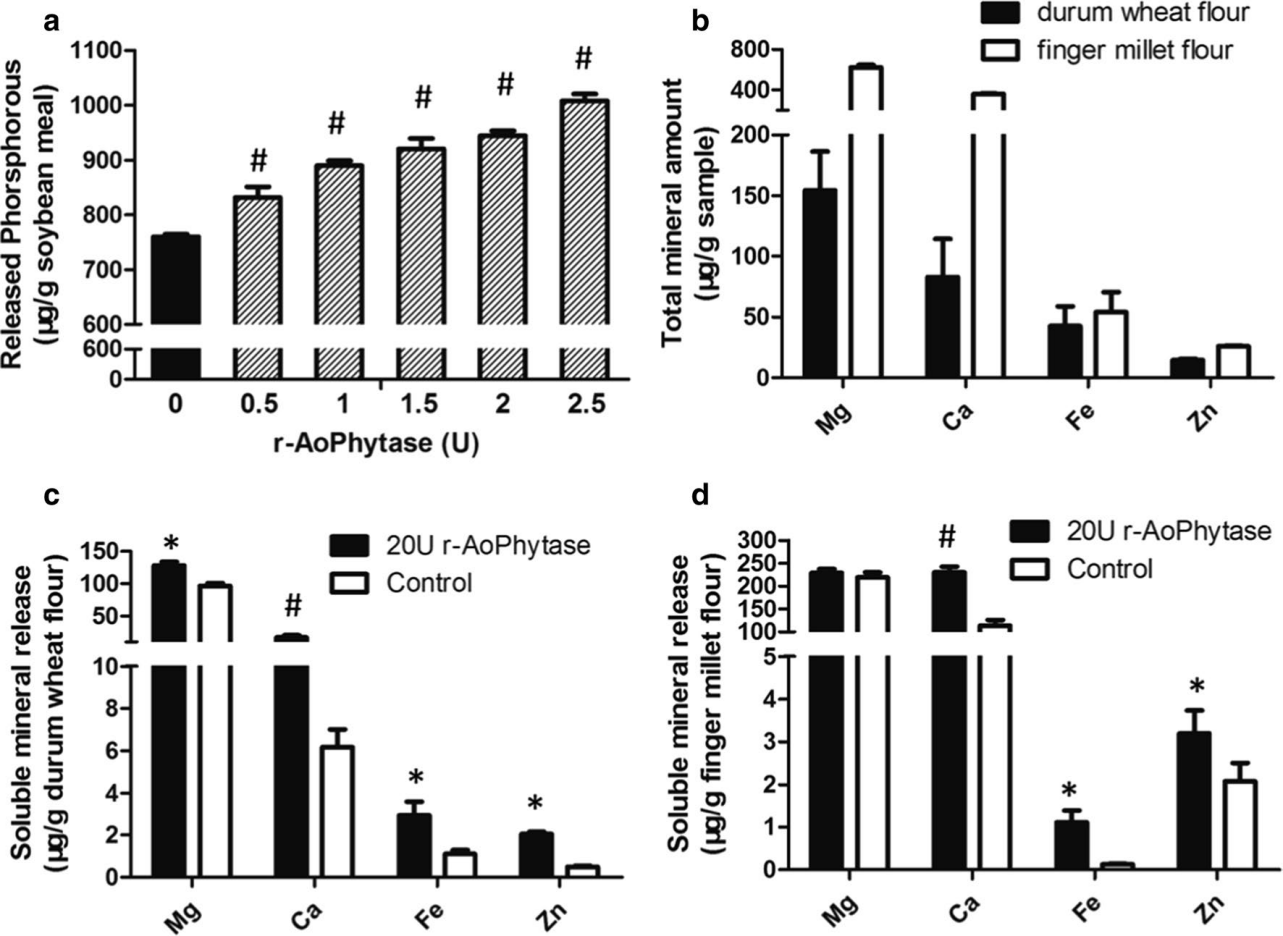
Effect of r-Aophytase on the release of inorganic phosphorus or minerals from feedstuffs. **a** With an increase of the amount of r-Aophytase, the release of inorganic phosphorus from soybean meal increases accordingly; **b** the total amount of the minerals, Ca^2+^ Fe^2+^, Zn^2+^ and Mg^2+^ in 1 g durum wheat flour or finger millet flour; **c** r-Aophytase significantly improved the release of Ca^2+^ Fe^2+^, Zn^2+^ and Mg^2+^ ions from durum wheat flour; **d** r-Aophytase significantly improved the release of Ca^2+^ Fe^2+^, Zn^2+^ and Mg^2+^ ions from finger millet flour. ^#^ P < 0.01, 0.5 U–2.5 U r-Aophytase vs. 0 U r-Aophytase, or 20 U r-Aophytase vs. Control; * P < 0.05, 20U r-Aophytase vs. Control

We finally explored the potential application of r-Aophytase in feed processing. We found that r-Aophytase significantly increased the release of inorganic phosphorus from soybean meal. As the amount of enzyme increases, the amount of phosphorus released increases accordingly (Fig. 7a). Similarly, we found that r-Aophytase significantly improved the release of Mg^2+^, Ca^2+^, Fe^2+^ and Zn^2+^ ions from durum wheat flour (Fig. 7c). Compared with the control, the release ratios were increased by 20.5%, 13.2%, 4.3%, and 10.5%, respectively. The release of soluble minerals from the finger millet flour was also evaluated. We found that r-Aophytase notably increased the release of Mg^2+^ ion and also significantly increased Ca^2+^, Fe^2+^ and Zn^2+^ ions from the finger millet flour (Fig. 7d). Compared with the control, the release ratios were increased by 1.6%, 32.1%, 1.9%, and 8.3%, respectively.

## Discussion

This work for the first time reports a novel fungalβ-propeller phytase, Aophytase, derived from a nematophagaous fungus *A. oligospora*. The optimal pH of r-Aophytase is about 7.5, which is beneficial to its utilization in the feed processing industry. The neutral to alkaline pH optimum of Aophytase presumably have potential application in the diets of aquaculture animals [10] and poultry [4]. The major supplements in the feed industry are derived from neutral pH phytate-rich plant meals such as cereals, legumes, soybean and others [10]. Aophytase is maybe a best choice for the processing of these types of feedstuffs. For instances, soybean processing requires additional phytase input or other treatment because endogenous soybean phytate with an optimal pH range of 4.5–5.0 is not highly active in the neutral pH processing environment of the raw materials. Thus, phytase with an alkaline pH optimum helped to degrade phytate during soybean processing without additional treatment [27]. Even in food industry, such as in whole-wheat bread preparation, the addition of BPP phytase DS11 with a pH range of 7.0–8.0 isolated from Bacillus amyloliquefaciens also successfully improved the mineral availability [28]. Furthermore, the optimum temperature of r-Aophytase is about 50 °C, which does not indicate the profile of its thermos stability. Whether it is stable enough through pelleting temperatures in the feed processing, it is dependent on its thermos stability, not the temperature optimum. We are determining the thermo tolerance of Aophytase in our laboratory to define the optimal temperature ranges in the different applications. Regardless of its thermo tolerance, Aophytase are presumably at least useful for the aquaculture because of their neutral gut pH and the lower body temperatures [22] or for the post pellet phytase application in feed processing [29]. Additional, if the thermo tolerance fails to meet the pelleting temperature requirement in the feed processing, it could be improved by encapsulation, or by changes in amino acid and modification of post-transcriptional glycosylation in different expression systems, or by employing random and rational designs for obtaining stable Aophytase variants [29, 30].

Compared with the traditional BPP phytase derived from bacteria, the recombinant fungal-derived Aophytase has higher specific enzyme activity. We speculate that the high specific enzyme activity of Aophytase should be related to the adaptive mechanism by which the fungus survives in the low nutrient environment. Given that Aophytase (G1XN29, Fig. 1d) was found to be only expressed in the parasitic phase of *A. oligospora* by comparison of the surface proteins isolated from 3D traps(+) with those of 3D traps(−), we speculate that Aophytase may be involved in the phase switch from saprophytic to parasitic state, resulting in an improvement of nematode-trapping activity and enhancement of the viability of the fungus in a nutrient-poor environment. This speculation is further substantiated by the structural analysis of Aophytase. We found that the protein has three functional domains following standard alignment analysis and 3D modeling. There are BPP type domains at the N-terminus and C-terminus, respectively, with two EGF-like domains in the middle. The C-terminal BPP-type domain is highly homologous to the *Bacillus*-derived 3-phytase (d1h6la), while the N-terminal BPP-type domain is homologous with the sonic hedgehog protein Hhip (c3ho5B) or human Wnt signaling co-receptor LRP6 (c3soqA) in a certain degree. The middle EGF-like domains share some homology with a human Wnt inhibitor Wif (c2ygqA). It is reported that d1h6la is a typically thermostable phytase with a six-bladed β-propeller structure belonging to the 3-phytase family. It is derived from *Bacillus* sp. strain DS11 and hydrolyzes phytic acid to 1,2,4,5,6-myo-inositol-pentakisphosphate [12]. The c3ho5B interacts with human sonic hedgehog, inhibiting the hedgehog signaling pathway. It is composed of two EGF domains and a six-bladed beta propeller domain, involved in the regulation of embryonic development [31]. The c3soqA is a human Wnt signaling co-receptor LRP6 (low-density lipoprotein receptor-associated protein 6), containing a β-propeller domain of YWTD-conserved motif. It acts as a co-receptor of Wnt signaling pathway to regulate Wnt/β-catenin with LRP5. This signaling pathway plays a very important role in embryonic development and disease development [32]. The c2ygqA is a human Wnt inhibitor Wif [33], also involved in human embryonic development and homeostatic regulation. It contains a typical Wif domain and an EFG-like domain. The EFG-like domain is homologous with AoPytase. Therefore, given that Aophytase is only present in 3D Trap(+) samples and this protein is not found in hyphae without 3D traps, we have reason to speculate that Aophytase not only has a typical bacterial-derived 3-phytase activity, but may be involved in the regulation of the cell growth and morphogenesis of *A. oligospora*, helped by its N-terminal BPP domain and the middle EGF-like domain.

Aside from the potential role of Aophytase in the development of the 3D traps of *A. oligospora*, this study focused on its recombinant expression, activity evaluation and potential application in feed processing. In order to obtain highly active phytase, we integrated codon-optimized Aophytase gene into the genome of *Pichia pastoris* GS115. We found that Aophytase was successfully secreted into the fermentation broth after 72-h induction with methanol. Since the r-Aophytase has a 6×His-tag, we used a nickel NTA affinity chromatography for its purification, and finally obtained r-Aophytase with electrophoretic purity. The specific enzyme activity of the purified Aophytase was further measured as 74.71 U/mg. In comparison with the bacterial-derived recombinant BPP phytase [21, 22, 24, 34–36] (Table 1), we found that the specific activity of r-Aophytase reported in this study is much higher than those of recombinant BPP phytases derived bacteria, such as *Bacillus*, *Janthinobacterium*, *Pedobacter*, *Serratia*, etc. It has been generally reported that BPP phytases from different resources showed relatively low catalytic turnover number. For example, the least Km value of 0.087 mM was so far described for the BPP phytase from *S. oneidensis* MR-1, however the Kcat (s^−1^) of 2.93 is also very low [4]. By contrast, the BPP phytase from *Pedobacter nyackensis* (PhyP) had a Km value of 1.28 mM, showing maximum Vmax value of 71.9 U mg^−1^ and accordingly maximum Kcat (s^−1^) of 45.1. The PhyP was then demonstrated its potential as an aquatic feed additive in the aquaculture industry due to relatively high turnover number (Kcat) and high activity at neutral pH and room temperature [22]. r-Aophytase (1.015 mM for Km, 72.46 U mg^−1^ for Vmax, and 13.57 s^−1^ for Kcat) in this work had similar catalytic kinetics compared to the PhyP from *P. nyackensis*. According to these studies, we conjecture that r-Aophytase, showing the neutral to alkaline pH optimum and a catalytic kinetics similar to the PhyP, at least has potential application in the diets of aquaculture animals.

## Conclusions

This paper reports a novel BPP phytase derived from *A. oligospora*, which is the first BPP phytase derived from fungi. Compared with the traditional BPP phytases derived from bacteria, r-Aophytase has higher specific enzyme activity with optimum pH of 7.5 and optimal temperature of 50 °C. r-Aophytase effectively improved the release of inorganic phosphorus and soluble minerals from feedstuffs. These favorable properties of r-Aophytase, including high specific enzyme activity, near-neutral optimal pH, relatively high optimal temperature, and enhancement of the release of inorganic phosphorus and soluble minerals, will potentiate its utilization in the feed processing industry in the future.

## Supplementary information

**Additional file 1: Figure S1.** Codon-optimized Aophytase gene (Genbank accession NO. MN688990) for *Pichia pastoris* expression. Note: GAATTC is *EcoR*I recognition site; GTCGAC is *Sal*I recognition site. **Figure S2.** Linearized pPICZαA-Aophytase by *Sac*I digestion. Plasmid pPICZαA-Aophytase was digested by *Sac*I, and detected by DNA agarose gel electrophoresis. (M) DL5000 DNA Marker; (1) plasmid pPICZαA-Aophytase; (2) Linearized pPICZαA-Aophytase shows a single band with its length more than 5000 bp in the agarose gel, well agreeing with its length of 5767 bp. **Figure S3.** The standard curve for Pi determination. One mL of AMES solution was added to 1ml phosphorus standard solution followed by 50 ºC water-bath for 20 min. The OD values of the reaction mixtures were determined at λ_700_ nm. The standard curve of OD_700_ v.s. phosphorus concentration was plotted. Through linear fitting and regression, the linear equation is y = 6.2417x + 0.0723 and R^2^ = 0.9971.

## Data Availability

Not applicable.
