# Supplementary material for: A novel fungal beta-propeller phytase from nematophagous Arthrobotrys oligospora: characterization and potential application in phosphorus and mineral release for feed processing
Source: Microb Cell Fact. 2020 Apr 6;19:84. doi: 10.1186/s12934-020-01346-9 (PMC7137328; doi:10.1186/s12934-020-01346-9)
Supplement: Supplementary file 1 — Additional file 1: Figure S1. Codon-optimized Aophytase gene (Genbank accession NO. MN688990) for Pichia pastoris expression. Note: GAATTC is EcoRI recognition site; GTCGAC is SalI recognition site. Figure S2. Linearized pPICZαA-Aophytase by SacI digestion. Plasmid pPICZαA-Aophytase was digested by SacI, and detected by DNA agarose gel electrophoresis. (M) DL5000 DNA Marker; (1) plasmid pPICZαA-Aophytase; (2) Linearized pPICZαA-Aophytase shows a single band with its length more than 5000 bp in the agarose gel, well agreeing with its length of 5767 bp. Figure S3. The standard curve for Pi determination. One mL of AMES solution was added to 1ml phosphorus standard solution followed by 50 ºC water-bath for 20 min. The OD values of the reaction mixtures were determined at λ700 nm. The standard curve of OD700 v.s. phosphorus concentration was plotted. Through linear fitting and regression, the linear equation is y = 6.2417x + 0.0723 and R2 = 0.9971. [file 12934_2020_1346_MOESM1_ESM.docx]

**Microbial Cell Factories**

A novel fungal beta-propeller phytase from nematophagous *Arthrobotrys oligospora*: characterization and potential application in phosphorus and mineral release for feed processing

Xianjuan Hou^1,2,3,†^, Zhen Shen^1,2,3,†^, Na Li^1,2,3^, Xiaowei Kong^1,2^, Kangliang Sheng^1,2^, Jingmin Wang^1,2^, Yongzhong Wang^1,2,3,4,^*

^1^ School of Life Sciences, Anhui University, Hefei 230601, Anhui, China.

^2^ Key Laboratory of Human Microenvironment and Precision Medicine of Anhui Higher Education Institutes, Anhui University, Hefei 230601, Anhui, China.

^3^ Anhui Key Laboratory of Modern Biomanufacturing, Hefei 230601, Anhui, China.

^4^ Institute of Physical Science and Information Technology, Anhui University, Hefei 230601, Anhui, China.

^†^ Xianjuan Hou and Zhen Shen contributed equally to this work.

* Correspondence: yzwang@ahu.edu.cn

GAATTCGCTGACAAGTTCTCCATCACTTTGCCAATTACTGCCAGAACCTCTTCCGTTGAATCTGACTCTGCTGCCGTTTACTACCCATCCAAGTCTAAGTACTCCCCAATCTTCATCGGTAACGACGGTTCTGCTGAAACTGGTGGTTTTCACGTCTACGAGTTGTACGGTAAGAGATCCGACGCCTTGGTCAAAGAATTGGGTGCTTACAAGACCGGTAGATCCAAGTTGGTTGAGGTTGTTTACGGTGAGGACAGAGACTTCGTTGTCACTTTGTCTATGTCCGACGGTATGTTCAGAGTGTTCGAGGTCGATGGTAAGAACGGTGTCAGAGGTTTGAAGGCCGAGAAGTTGGTCAGAGGTGATTTCTCCGCTATGTGTACCTGGAAGTCTAAGGTTGGTGAGTACGTCTACGTCCTGGGTAAAAGATGGGGTTACAGATTCTTGGTCCGTGAGAAGAAAGGTGGTAGAGGTGTTGAGGTCGTTCAGACTCAAGAGTTCGGTATTCCAATCGAGCCAAACTCCTGTACTGTTTCCCCAGAAGGTAAGGTTTTCTTGGCTGGTGACTCCGGTAAGGTGTTTTCTTTTTTGGCCGTTGACGAGACTGCTGCCCCAAAGATAGTTGAAGTTGGTGAACTTGGTGGTGGTGACGAGGTTAAGGGTTTGAAGATCTACCACGGTAAGAAGGACACCTACTTGTTGGTTGGTTTGGAGGACGGTATCGAGGTGTTCGACATTAAGAAGTTGGGATCCTCCTTGGGTAAGATCCAATTCGACGACGAAGAGTTGGAAGTCGGTGATTTCGCTGTTCATCAGACTTCCGCTAAGGGTTACGAAGATGGTTCCATCGTTTTCGCTGGTGAAGATGGTGAGGGAAAGTTCTTCGGTGTTTCCTCCTTGACTCCTCTGTTCAAGGCTCTTGGTAAGGGTAAGCTGAACACCAAGTACGACCCAAGAGACTGTACTGACAACCACGCTAGACCAAAGAAGTGTGCTAACTTGTCCGACTGCAACGGTTACGGTTACTGTCCAAAGGACTCCAGAGACAAGAAGGCTACTTGCGACTGTTTTCCAGGTTTGACCGGTAAGACCTGCAACAAGATTACCTGTCCATCCAACTGCACTTCCCCATCTCATGGTACTTGTACCGGTCCAAACATCTGCACTTGTATCCCACCATTCACTGGTGAGAACTGTGCTACTTTGGCTGTTCCAGCTAGATACGAGACTGAAGAATCTGGTGGTGCTGATGGTGATGACCCAGCTATTTGGATTCACCCAACTGACAAGACCAAGTCCAGAATCATCACCACCGTTAAGTCCGAAGTTGGTTCCGGTTTGGGTGTCTACGATCTGAAGGGTAAGAGAACCGGTGGTGTTTCAGGTGGTGAACCTAACAACGTTGACGTCTTGTACGGTGTTGAGTTCGCCGGTAGAAAGGTTGACTTGGCTGTTGCTGCTTGTAGAGCTGACGACACCATCTGTATCTACGAGATCACTCCAACCGGTGACTTGGTTACTATTCCAGGTGGTGTTCAACCATTGCCACCAGCTGTCAAAGAGCTGGAAAAGAAGTTCAAGGTCTACGGTTCCTGCGTCTACCACTCTCCAAAGACTGGTGCTTACCACATCTTCGTCAACTCCAAGTCCTCCTTGTACTTGCAGTTCCAGTTGTCCGCTACTACTGACGGAAAGTTGAACACTACCCTGGTCAGACACTTCTACGCTGGTAACAGAGGTCAAGTTGAGGGTTGTGTTGTTGACGACGAAAACTCTTCCCTGTTCTTGGGTGAAGAACCATACGGTATTTGGTCCTACGATGCTGAACCAGACCAACCTGCTGTTGGTACTTTGGTTGACAACACTGTCGTTGACGGTGGTAAGTTGCACGCTGATGTTGAGGGTGTTACCTTGGTTTACGGAAAGACCAAGAAAGAGGGTTACATCATCGTTTCTTGCCAGGGTTACTCCGAGTACAACATCTACCAAAGATACCCACCACACGAGTTCGTCATGTCCTTCTCTATCCCTGACAACAAAGAGAAGGGCGTTGACAGAGTTACCAACACTGACGGTATTACTGCCGTTGGTGCCAACTTGGGTAAAGAATGGCCATACGGAATGGTTGTTGTCCACGACGATGTTAACGAAGCTGCTGGTGGTGGCGTTAGAGCTGATGCTACTTTTAAGATTGTCGGTCTGGGTGACATCTTGGGTAACAAGGCTGTGAAAGAGTTGGGTTTGCTGAAGGGTGTTGATGAGAACTGGGACCCAAGAAAGGTCGAC

**Figure S1** Codon-optimized Aophytase gene (Genbank accession NO. MN688990) for *Pichia pastoris* expression. Note: GAATTC is *EcoR*I recognition site; GTCGAC is *Sal*I recognition site.


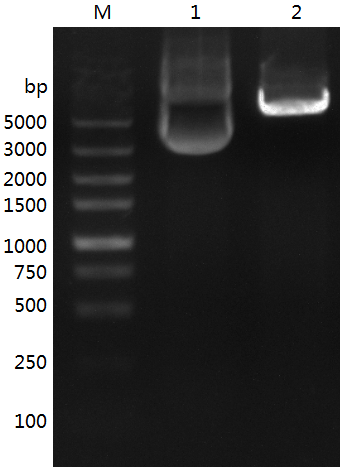


**Figure S2** Linearized pPICZαA-Aophytase by *Sac*I digestion. Plasmid pPICZαA-Aophytase was digested by *Sac*I, and detected by DNA agarose gel electrophoresis. (M) DL5000 DNA Marker; (1) plasmid pPICZαA-Aophytase; (2) Linearized pPICZαA-Aophytase shows a single band with its length more than 5000bp in the agarose gel, well agreeing with its length of 5767bp。


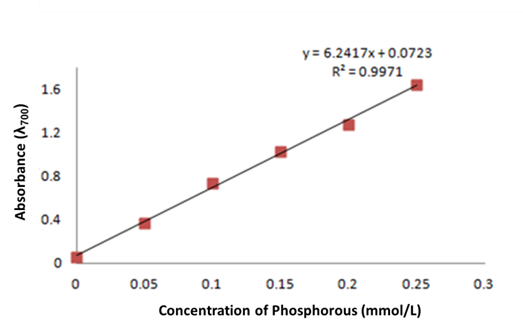


**Figure S3** The standard curve for Pi determination. One ml of AMES solution was added to 1ml phosphorus standard solution followed by 50ºC water-bath for 20 min. The OD values of the reaction mixtures were determined at λ_700_ nm. The standard curve of OD_700_ v.s. phosphorus concentration was plotted. Through linear fitting and regression, the linear equation is y=6.2417x+0.0723 and R^2^=0.9971.
